# Supplementary material for: MetaRibo-Seq measures translation in microbiomes
Source: Nat Commun. 2020 Jun 29;11:3268. doi: 10.1038/s41467-020-17081-z (PMC7324362; doi:10.1038/s41467-020-17081-z)
Supplement: Supplementary file 10 — Supplementary Data 7 [file 41467_2020_17081_MOESM10_ESM.zip › File2/Confidence_High_Taxonomy/218297_out.krona.html]

Javascript must be enabled to view this page.

members
magnitude
magnitudeUnassigned
count
unassigned
taxon
rank

218297\_out

2

2
superkingdom
2

976
phylum
1

200643
1
class

order
1
171549

1
family
171552

838
1
genus


SRS148676\_contig\_number\_8885
28137
1
species

1239
1
phylum

186801
1
class

order
1
186802

family
1
541000

946234
1
genus

1
species
1193534

SRS097920\_contig\_number\_5524
